# ChIP-seq Profiling Identifies Histone Deacetylase 2 Targeting Genes Involved in Immune and Inflammatory Regulation Induced by Calcitonin Gene-Related Peptide in Microglial Cells

**DOI:** 10.1155/2020/4384696

**Published:** 2020-08-04

**Authors:** Xingjing Guo, Dan Chen, Shuhong An, Zhaojin Wang

**Affiliations:** ^1^Department of Human Anatomy, Shandong First Medical University & Shandong Academy of Medical Sciences, Taian, China; ^2^Department of Physiology, Shandong First Medical University & Shandong Academy of Medical Sciences, Taian, China; ^3^Experimental Center, Shandong University of Traditional Chinese Medicine, Jinan, China

## Abstract

Calcitonin gene-related peptide (CGRP) is a mediator of microglial activation at the transcriptional level. The involvement of the epigenetic mechanism in this process is largely undefined. Histone deacetylase (HDAC)1/2 are considered important epigenetic regulators of gene expression in activated microglia. In this study, we examined the effect of CGRP on HDAC2-mediated gene transcription in microglial cells through the chromatin immunoprecipitation sequencing (ChIP-seq) method. Immunofluorescence analysis showed that mouse microglial cells (BV2) expressed CGRP receptor components. Treatment of microglia with CGRP increased HDAC2 protein expression. ChIP-seq data indicated that CGRP remarkably altered promoter enrichments of HDAC2 in microglial cells. We identified 1271 gene promoters, whose HDAC2 enrichments are significantly altered in microglia after CGRP treatment, including 1181 upregulating genes and 90 downregulating genes. Bioinformatics analyses showed that HDAC2-enriched genes were mainly associated with immune- and inflammation-related pathways, such as nitric oxide synthase (NOS) biosynthetic process, retinoic acid-inducible gene- (RIG-) like receptor signaling pathway, and nuclear factor kappa B (NF-*κ*B) signaling pathway. The expression of these key pathways (NOS, RIG-I, and NF-*κ*B) were further verified by Western blot. Taken together, our findings suggest that genes with differential HDAC2 enrichments induced by CGRP function in diverse cellular pathways and many are involved in immune and inflammatory responses.

## 1. Introduction

Microglia are innate immune cells of the central nervous system that are responsible for the excessive and chronic neuroinflammatory response following damage and disease [1, 2]. Calcitonin gene-related peptide (CGRP) as a mediator of glial cell activation plays a role as signaling molecule, mediating interactions between damaged neurons and surrounding glial cells [3]. Previous research has demonstrated that the presence of CGRP induced the activation of microglia at the transcriptional level through expression of the immediate-early genes in cultured microglia [4, 5].

CGRP operates via its receptor that, in many cell types, leads to a range of biological effects including those associated with neurons and microglia [6, 7]. A functional CGRP receptor consist of 3 components, the calcitonin receptor-like receptor (CRLR), receptor activity-modifying protein 1 (RAMP1), and CGRP receptor component protein (CRCP) that couple the receptor to the cellular signal pathway leading to increased intracellular cAMP and activated protein kinase A (PKA) [8]. Accumulating evidence suggested that microglia express functional CGRP receptors, as treatment with CGRP triggers microglial activation by induction of c-fos gene expression, cAMP accumulation, and release of proinflammatory mediators in microglia [4, 5, 9]. A previous study indicated that CGRP may act as a paracrine mediator to induce activation of glial activation [10, 11]. Administration of CGRP in rat models of temporomandibular joint disorder contributed to the increased microglial marker of OX-42 expression and microglial activation in the spinal cord [12], suggesting a functional link between CGRP and microglial activation.

Histone deacetylase (HDAC)1/2 are essential for microglial survival and expansion during development [13]. Increased activity of HDAC2 is closely related to glial activation in a mouse model of retinal injury [14]. Selective inhibition of HDAC1 or 2 suppresses the expression of inflammatory cytokines in BV2 murine microglia activated with lipopolysaccharide (LPS) [15]. Previous data indicated that HDAC2 activates NF-*κ*B and promotes NF-*κ*B-dependent gene expression [16], which plays a crucial role in microglial activation [17].

The ability of CGRP to activate microglial cells at the level of transcription raises the question of whether the gene expression induced by CGRP associates with epigenetic regulation by HDAC2 in activated microglia. In the present study, we adopted chromatin immunoprecipitation followed by sequencing (ChIP-seq) to profile and compare the variation of HDAC2 enrichments in target genes at the genome-wide level in the microglial cell line (BV2) from treatment of CGRP and control to gain a better understanding of a potential role for this peptide in the responses of microglia. In addition, the expression of CGRP receptor components was also explored in BV2 cells and Western blot was used to determine whether the expression of HDAC2 in microglia was influenced by CGRP, hoping that these studies could further understand the underlying regulatory mechanism of microglial activation by CGRP at the molecular level.

## 2. Materials and Methods

### 2.1. Cell Culture and Drug Administration

The mouse microglial cell line (BV2) was obtained from the Cell Bank of the Chinese Academy of Sciences (Beijing, China). Microglial cells were cultured in Dulbecco's modified Eagle's medium (DMEM, Gibco) supplemented with 10% fetal bovine serum (FBS, Biological Industries) incubated at 37°C in an atmosphere of 5% CO_2_. BV2 cells continuously stimulated with CGRP peptide (1 *μ*mol/L, Tocris Bioscience, Cat. #1161) at 1, 2, 4, and 6 h, respectively. Cells without CGRP peptide were used as the control. To assess the possible impact of HDAC2 on the expression of iNOS, RIG-I, and NF-*κ*B induced by CGRP, 0.5 mmol/L valproic acid (VPA, HDAC2 inhibitor, MedChemExpress, Cat. #1069-66-5) were preapplied for 60 min and coapplied together with CGRP for 2 h at 37°C.

### 2.2. Immunofluorescence of Cultured Cells

Immunofluorescence was performed essentially as described before [18]. BV2 cells were cultured on poly-L-lysine-coated coverslips in six-well plates. Following a single wash in phosphate-buffered saline (PBS), cultured microglial cells were fixed in 4% paraformaldehyde for 15 min at room temperature. Double-labeling immunofluorescence staining for primary antibodies against ionized calcium binding adaptor molecule 1 (Iba1, a marker for microglia; 1 : 200, Novus, Cat. #NB 100-1028) and CRLR (1 : 200, Abcam, Cat. #ab84467), RAMP1 (1 : 200, Sigma-Aldrich, Cat. #SAB250086), or CRCP (1 : 200, Proteintech, Cat. #14348-1-AP) on coverslip-cultured microglia was performed. Coverslips were incubated with a mixture of the two primary antibodies overnight. Following three washes with tris-buffered saline (TBS), coverslips were treated with a mixture of matching secondary antibodies (Jackson ImmunoResearch). The specificity of antibodies used was checked by Western blotting and/or omission of the primary antibodies. No specific immunoreactivity was detected in these controls.

### 2.3. Chromatin Immunoprecipitation

Chromatin was prepared from fixed mouse microglial cells (stimulated with 1 *μ*mol/L CGRP, 2 h) and sonicated fragments ranged in size from 200 to 1500 bp. Approximately 2 × 10^7^ cell equivalents were used for each immunoprecipitation. ChIP was performed as described previously [19], using anti-HDAC2 antibody (Abcam, Cat. #ab12169, ChIP Grade) or a control rabbit IgG.

### 2.4. Sequencing Library Preparation, Cluster Generation, and Sequencing

DNA samples were end-repaired, A-tailed, and adaptor-ligated using TruSeq Nano DNA Sample Prep Kit (Cat. #FC-121-4002, Illumina), following the manufacturer's instructions. ~200-1500 bp fragments were size-selected using AMPure XP beads. The final size of the library was confirmed by Agilent 2100 Bioanalyzer. Samples were diluted to a final concentration of 8 pmol/L, and cluster generation was performed on the Illumina cBot using HiSeq 3000/4000 PE Cluster Kit (Cat. #PE-410-1001, Illumina), following the manufacturer's instructions. Sequencing was performed on Illumina HiSeq 4000 using HiSeq 3000/4000 SBS Kit (300 cycles) (Cat. #FC-410-1003, Illumina), according to the manufacturer's instructions.

### 2.5. Data Collection and ChIP-seq Analysis

After sequencing platform-generated sequencing images, stages of image analysis and base calling were performed using Off-Line Basecaller software (OLB V1.8). Sequence quality was examined using the FastQC software. After passing Solexa CHASTITY quality filter, clean reads were aligned to mouse genome (UCSC MM10) using Bowtie software (V2.1.0) [20]. Aligned reads were used for peak calling of ChIP regions using MACS V1.4.2 [21]. Statistically significant ChIP-enriched regions (peaks) were identified by comparison of IP vs. input or comparison to a Poisson background model, using a *P* value threshold of 10^−4^. Peaks in samples were annotated by the nearest gene using the newest UCSC RefSeq database [22]. The annotation of the peaks which were located within -2 kb to +2 kb around the corresponding gene TSS in samples can be found from the peaks-promoter-annotation.

### 2.6. Bioinformatics Analysis

The Gene Ontology (GO) functional and Kyoto Encyclopedia of Genes and Genomes (KEGG) pathway enrichment analyses were performed using the Database for Annotation, Visualization and Integrated Discovery (DAVID) and KEGG Orthology-Based Annotation System (KOBAS) online tools (http://www.geneontology.org and http://www.genome.jp/kegg) [23, 24].

### 2.7. Western Blotting

Western blot analysis was performed as described before [18]. Cultured microglial cells were lysed, and the protein was extracted. The protein lysate from each sample was separated electrophoretically on a 10% sodium dodecyl sulfate-polyacrylamide gel and then transferred to a polyvinylidene fluoride (PVDF) membrane. After blocking with 5% nonfat milk in TBS-T (containing 0.1% Tween-20) for 2 h, membranes were incubated with HDAC2 (Abcam, Cat. #ab32117), NF-*κ*B (Abcam, Cat. #1559-1), RIG-I (Abcam, Cat. #ab45428), and iNOS (Abcam, Cat. #ab3523) in 5% nonfat milk in TBS-T overnight at 4°C. After washes with TBS-T, membranes were incubated with appropriate secondary antibodies for 2 h. Results were visualized using an ECL chemiluminescence system. GAPDH rabbit mAb antibody (Cell Signaling Technology, Cat. #2118) was also used as a probed control to ensure the loading of equivalent amounts of sample proteins. Band densities were compared in TotalLab software (version 2.01; Bio-Rad, Hercules, CA).

### 2.8. Statistical Analysis

Data are presented as the mean ± standard error of the mean (SEM). For the analyses of Western blot data, Mann-Whitney *U* tests were used for comparisons between two groups, and Kruskal-Wallis tests with Dunn's multiple comparisons post hoc tests were used for comparisons among multiple groups. The data from the Rotarod test were compared using Kruskal-Wallis tests with Dunn's multiple comparisons post hoc tests. Significance was defined by *P* values < 0.05.

## 3. Results

### 3.1. CGRP Increases HDAC2 Expression in Microglial Cells

**Figure 1:**
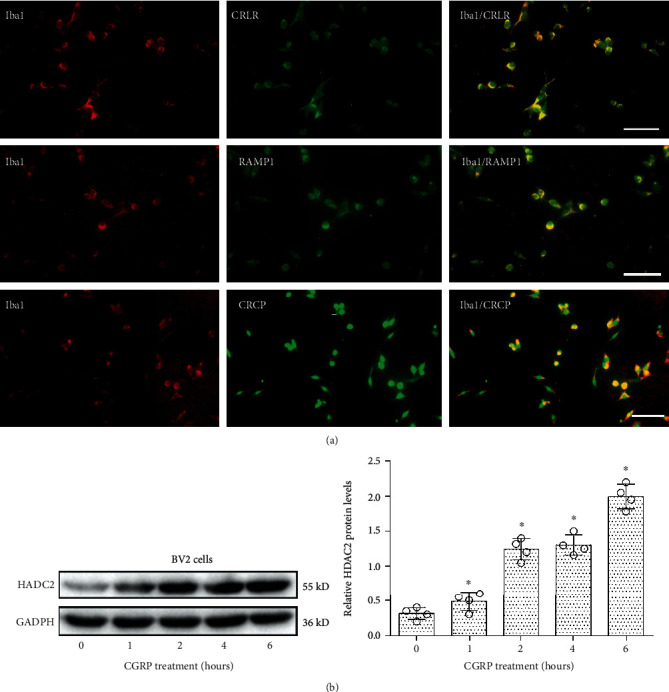
Cultured mouse microglial cells (BV2) expressed Iba1 and CGRP receptor components CRLR, RAMP1, or CRCP. (a) Expression of Iba1 (a marker of microglia, red) and its colocalization with CRLR, RAMP1, or CRCP staining (green) in cultured microglial cells. Scale bar 40 *μ*m. (b) Western blot analysis of HDAC2 expression in microglia with treatment with CGRP at 0, 1, 2, 4, and 6 h, respectively. Relative amounts of proteins were calculated by normalizing to GAPDH. Data are presented as the mean ± SEM (*N* = 4 independent cell culture preparations, Mann-Whitney *U* tests or Kruskal-Wallis tests). ^∗^*P* < 0.05.

To study the effect of CGRP on microglial cells, we first investigated the expression of CGRP receptor components on microglia.  Figure 1(a) shows an example of colocalization of CRLR, RAMP1, and CRCP with the Iba1 (microglial marker) immunoreactivity on microglial cells in culture. Nearly all of the Iba1-positive cells expressed CGRP receptor components CRLR, RAMP1, and CRCP.

The expression of HDAC2 protein in microglia was assessed by Western blot following treatment with CGRP for 0, 1, 2, 4, and 6 h, respectively. As shown in Figure 1(b), CGRP treatment significantly increased the expression of the HDAC2 protein level in microglia (*P* < 0.05). CGRP was found to induce the expression of HDAC2 in a time-dependent manner with a maximal effect observed after 6 h.

### 3.2. Genome-Wide Profile of HDAC2 Targets in Microglia after CGRP Treatment

To investigate the role of HDAC2 on microglia after treatment with CGRP, the profile of HDAC2 targets in microglial cells was analyzed using an Illumina HiSeq 4000 sequencing technique after stimulation with CGRP for 2 h. Model-based Analysis of ChIP-seq (MACS, v1.4.2) software was used to detect the ChIP-enriched regions (peaks) from ChIP-seq data. Differentially enriched regions with statistical significance between the CGRP-treated group and the control were identified by Detecting Differential Chromatin Modification Sites from ChIP-seq Data with Biological Replicates (diffReps), cut-off: FC = 2.0, *P* = 10^−4^).

**Figure 2:**
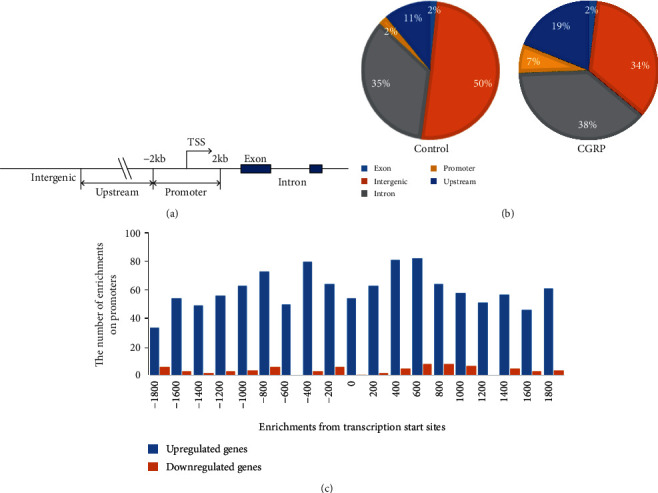
Effect of CGRP on the enriched region (peak) distribution of the ChIP-seq reads of HDAC2 in microglia treated with CGRP compared with the control. (a) Genome-wide distribution of enrichments relative to annotated genes. (b) The peak distribution of ChIP-seq reads of HDAC2 in control and microglial cells treated with CGRP. (c) The distribution of HDAC2 peaks on promoters relative to gene transcription start sites (TSSs). Shown are HDAC2 peak frequencies relative to the distance from the nearest annotated TSS in microglial cells treated with CGRP.

The ChIP-seq for the CGRP-treated microglial cells generated 21827 enriched regions (peaks), including 17646 up peaks and 4181 down peaks, compared with the control group. The peak distribution of ChIP-seq reads of HDAC2 was showed in Figures 2(a) and 2(b). We identified 1271 gene promoters, whose HDAC2 enrichments are significantly altered in microglial cells treated with CGRP, including 1181 upregulated genes and 90 downregulated genes (Table S1). The distribution of HDAC2-enriched promoters was mapped to proximal regions of transcription start sites (TSSs) of RefSeq genes (from about -1800 bp to +1800 bp of TSSs, Figure 2(c)).

### 3.3. GO Analysis of Peaks Relative to Annotated Genes

**Figure 3:**
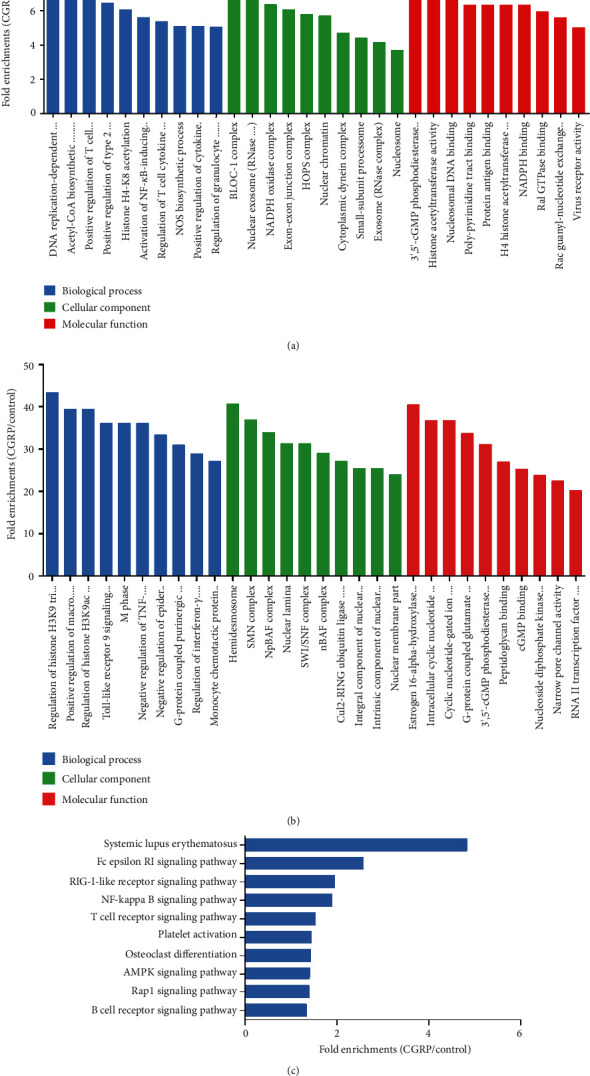
The Gene Ontology (GO) and Kyoto Encyclopedia of Genes and Genomes (KEGG) pathway analyses of genes with differentially enriched HDAC2 in microglial cells treated with CGRP. GO annotation of upregulated genes (a) or downregulated genes (b) with HDAC2 enrichments of the CGRP treatment group versus the control. Bar plots show the top ten fold enrichment values of the significant enrichment terms involving biological process (BP), cellular component (CC), and molecular function (MF). (c) KEGG pathway analysis of genes with differentially enriched HDAC2 in microglial cells treated with CGRP. The bar plot shows the top ten fold enrichment values of the significant enrichment pathway. Analysis by DAVID and KOBAS online tools (http://www.geneontology.org and http://www.genome.jp/kegg).

To further understand functions of annotated genes related to peaks, they were functionally classified using GO terminology. According to the functional annotation in GO database, key upregulated HDAC2 target genes were mostly enriched for biological process (BP) terms associated with positive regulation of T cell cytokine production (e.g., tumor necrosis factor receptor associated factor 6 (TRAF6), mucosa-associated lymphoid tissue lymphoma translocation gene 1 (MALT1)), histone acetylation (e.g., lysine acetyltransferase 8 (KAT8), KAT8 regulatory NSL complex subunit 2 (KANSL2)), activation of NF-*κ*B-inducing kinase activity (e.g., ZFP91 zinc finger protein (ZFP91), TRAF6, and MALT1), and NOS biosynthetic process (e.g., Fc fragment of IgE receptor II (FCER2A), nucleotide binding oligomerization domain containing 2 (NOD2), prostaglandin E receptor 4 (PTGER4), and toll-like receptor 2 (TLR2)). When focusing on cellular components (CC), the most represented categories were BLOC-1 complex, nuclear exosome (RNase complex), and NADPH oxidase complex. The most represented categories for molecular function (MF) were related to 3′,5′-cGMP phosphodiesterase activity, histone acetyltransferase activity, nucleosomal DNA binding, and NADPH binding. Most genes are well-known immune and inflammatory responses (e.g., TRAF6, TLR2, and FCER2A). Association with immune- and inflammation-related genes seems therefore to be a feature of CGRP-mediated HDAC2 enrichments. GO enrichment terms of BP, CC, and MF for upregulated genes are shown in Figure 3(a).

Meanwhile, key dowregulated HDAC2 target genes were enriched in BP terms such as regulation of histone H3K9 acetylation, toll-like receptor 9 signaling pathway, astrocyte activation, and negative regulation of tumor necrosis factor signaling. The most represented categories for CC were related to SMN complex and nuclear lamina and MF terms such as cyclic nucleotide-gated ion channel activity and cGMP binding. GO enrichment terms of BP, CC, and MF for downregulated genes are shown in Figure 3(b).

### 3.4. KEGG Pathway Analysis of Peaks Relative to Annotated Genes

KEGG pathway enrichment analysis was performed using the software KOBAS. The *P* < 0.05 was set as the threshold of significant enrichment. Based on the KEGG pathway enrichment analysis, key upregulated genes were significantly enriched in 10 signaling pathways, such as the Fc epsilon RI signaling pathway, RIG-like receptor signaling pathway, NF-*κ*B signaling pathway, and T cell receptor signaling pathway, which were mostly related to immune and inflammatory responses (Figure 3(c)). From these data, KEGG enrichment analysis related to immune and inflammatory responses accounted for the enrichment score which also agree with the GO enrichment analysis above. However, none of downregulated genes was significantly enriched in any KEGG pathway.

### 3.5. Validation of Key Pathways from ChIP-seq by Western Blot

**Figure 4:**
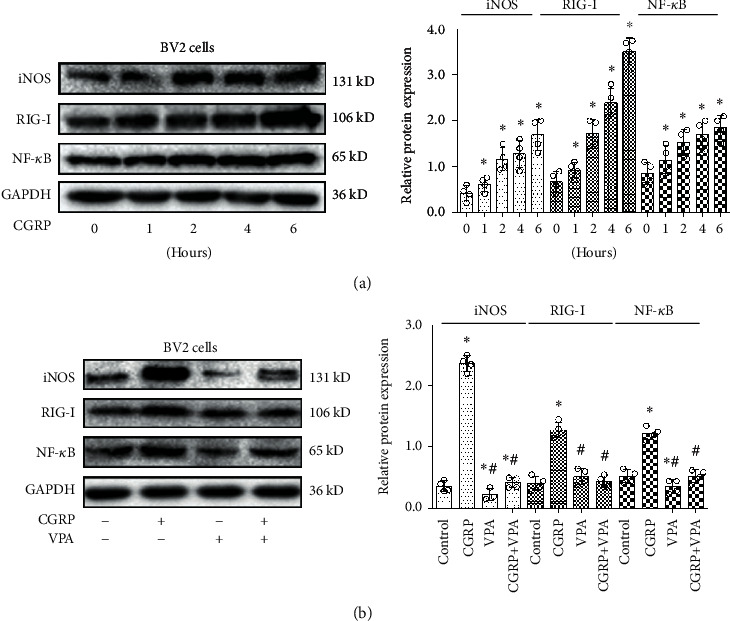
CGRP evokes increases in the expression of iNOS, RIG-I, and NF-*κ*B in microglial cells in a time-dependent manner. (a) Western blot analysis of iNOS, RIG-I, and NF-*κ*B expression in microglial cells with treatment of CGRP at 0, 1, 2, 4, and 6 h, respectively. (b) Western blotting analyses for the effect of HDAC2 inhibitor VPA on CGRP-evoked iNOS, RIG-I, and NF-*κ*B protein expression in microglial cells with treatment of CGRP and pretreatment with VPA. Relative amounts of proteins were calculated by normalizing to GAPDH. All values are expressed as the means ± SEMs (*N* = 4 independent cell culture preparations, Mann-Whitney *U* tests or Kruskal-Wallis tests). ^∗^*P* < 0.05 versus controls; ^#^*P* < 0.05 versus CGRP only groups.

In order to explore whether or not CGRP involves the NOS biosynthetic process, RIG-like receptor signaling pathway, and NF-*κ*B signaling pathway, the expression of iNOS, RIG-I, and NF-*κ*B protein levels in microglial cells was assessed by Western blot after treatment with CGRP for 0, 1, 2, 4, and 6 h, respectively. As shown in Figure 4(a), CGRP treatment significantly increased the expression of iNOS, RIG-I, and NF-*κ*B protein levels in microglia (*P* < 0.05). CGRP was found to induce the expression of iNOS, RIG-I, and NF-*κ*B in a time-dependent manner with a maximal effect observed after 6 h which was well in agreement with the HDAC2 enrichment analysis observed by bioinformatics analysis. The results of Western blot analyses provided evidence that the ChIP-seq method for large-scale gene expression quantification was reliable.

### 3.6. HDAC2 Inhibitor Suppresses the Increase of iNOS, RIG-I, and NF-*κ*B Expression Induced by CGRP

A recent report showed that HDAC2 increased the expression of iNOS in macrophages following inflammatory stimulation [25]. To test whether HDAC2 was involved in CGRP-induced expression of iNOS, RIG-I, and NF-*κ*B in this study, a HDAC2 inhibitor VPA was coapplied together with CGRP. As shown in Figure 4(b), CGRP increased the expression of iNOS, RIG-I, and NF-*κ*B following treatment of microglia with CGRP, and the HDAC2 inhibitor VPA partially or completely blocked the CGRP-mediated upregulation of iNOS, RIG-I, and NF-*κ*B (*P* < 0.05).

## 4. Discussion

Here, we mapped HDAC2 enrichment profiles induced by CGRP at these loci using ChIP-seq in mouse microglial cells and examined the influence of CGRP on HDAC2 enrichment profiles on promoter regions to test the hypothesis that the gene expression induced by CGRP was associated with HDAC2-mediated epigenetic regulation in microglia [15, 17]. ChIP-seq data showed that CGRP could substantially change HDAC2 enrichments on gene promoters, including 1181 upregulated genes and 90 downregulated genes, suggesting that CGRP could alter enrichment profiles of HDAC2 at promoters in a gene-specific manner.

HDAC2 is involved in regulating microglial activation and gene transcription [14, 15]. Our results showed that almost all cultured microglial cells expressed CGRP receptor components CRLR, RAMP1, and CRCP. Treatment of microglial cells with CGRP also increased the HDAC2 protein level. These results suggested that CGRP operated via its receptor that might contribute to modulation of gene transcription through HDAC2-medated epigenetic mechanism [26]. In order to obtain insights into HDAC2 target gene function, GO and KEGG analysis annotations were applied to the HDAC2 target gene pool. GO terms for BP process categories showed that key higher genes related with positive regulation of T cell cytokine production, NOS biosynthetic process, and activation of NF-*κ*B-inducing kinase activity. KEGG pathways for upregulated genes were mainly related to the Fc epsilon RI signaling pathway, RIG-like receptor signaling pathway, and NF-*κ*B signaling pathway. The expression of NOS, RIG-I, NF-*κ*B was further verified by Western blot in microglia following CGRP treatment. Therefore, alterations of this histone deacetylase induced by CGRP could have severe consequences on microglial activation at the level of transcription [5].

HDAC2 has been shown to regulate microglial activation and induce cytokine expression through the NF-*κ*B pathway [15, 17]. Previous data indicated that HDAC2 activates NF-*κ*B and promotes NF-*κ*B-dependent gene expression [16], which plays a crucial role in microglial activation [17]. NF-*κ*B can interact with corepressors HDAC1 and HDAC2 to regulate gene transcription [27, 28]. Activation of NF-*κ*B seems essential for the transcription of most of the proinflammatory molecules, such as cytokines and chemokines [29]. A previous report showed that activation of NF-*κ*B regulates microglial conversion to a proinflammatory type [30]. In the present study, we found CGRP increased the expression of HDAC2 and NF-*κ*B in microglia, suggesting that they might coordinately regulate the gene transcription for microglial activation induced by CGRP.

CGRP has been reported to activate microglia directly to produce proinflammatory mediators, including NOS and cytokines [31–33]. The specific recruitment of HDAC2 to NF-*κ*B at target promoters and the consequent effects on acetylation status may play an important role in regulating iNOS as well as other NF-*κ*B-dependent genes involved in inflammation [27, 34]. Activation of microglia displays NO production via iNOS activity which upregulates microglial phagocytosis and increases TRPV2 expression [35]. Our experiment showed that CGRP increased the expression of iNOS, NF-*κ*B, and RIG-I protein levels in microglia after CGRP treatment. Nonetheless, these increases in iNOS, NF-*κ*B, and RIG-I expression were inhibited by HDAC2 inhibitor, suggesting that increases of iNOS, NF-*κ*B, and RIG-I expression in microglia by CGRP might be associated with HDAC2. Furthermore, activation of RIG-I may induce the expression of proinflammatory cytokines through the activation of NF-*κ*B [36, 37]. A previous study demonstrated that HDAC2 inhibitor VPA reduced induction of RIG-I expression in different cancer cell lines by decitabine [38], suggesting that HDAC2 might be involved in regulation of RIG-I expression. Taken together with our results, these indicate that the upregulation of HDAC2 by CGRP may contribute to immune and inflammatory responses through the NO/iNOS, NF-*κ*B, and RIG-I signal pathways in microglial activation [39].

## 5. Conclusions

In summary, we systematically evaluated CGRP-mediated HDAC2 enrichments in microglial cells and gained new insights into links between key pathways and HDAC2 induced by CGRP in the microglial activation. Genomic analyses suggested that genes with the differential HDAC2 enrichments induced by CGRP function in diverse cellular pathways and many are involved in immune and inflammatory responses. However, further studies are needed to confirm our results.

## Data Availability

The data used to support the findings of this study are available from the corresponding authors upon request.

## Supplementary Materials

Supplementary Materials Table S1: differentially enriched regions for up and down peak-promoter-annotation.
